# Organometallic Chemistry of Propargylallenes: Syntheses, Reactivity, Molecular Rearrangements and Future Prospects

**DOI:** 10.3390/molecules29235670

**Published:** 2024-11-29

**Authors:** Michael J. McGlinchey

**Affiliations:** School of Chemistry, University College Dublin, Belfield, D04 V1W8 Dublin 4, Ireland; michael.mcglinchey@ucd.ie

**Keywords:** allenes, alkynes, thermolyses, molecular rearrangements, noble metal catalysis, cycloadditions, fluorenylidenes

## Abstract

Alkynylallenes offer the varied reactivity patterns of two different multiple bond linkages either separately or in concert. Initially, a short overview of their syntheses, structures, rearrangement mechanisms and synthetic utility, especially when treated with transition metal reagents such as gold(I), silver(I), platinum metals or metal carbonyls, is presented. Subsequently, we focus on the particular case of 1,2-dien-5-ynes (propargylallenes), whereby the shortness of the single atom bridge, and the consequent proximity of the allenyl and alkynyl moieties, facilitates metal-mediated interactions between them. It is shown how these metals can coordinate to either the alkyne or the allene fragment, thus leading to different cyclisation or rearrangement products, dependent also on whether it is the proximal or the distal double bond of the allene that participates in the reaction. Dimerisation of bromo-substituted fluorenylideneallenes bearing silyl or ferrocenyl substituents can occur in either head-to-head or head-to-tail fashion, thereby yielding propargylallene derivatives that undergo unexpected and novel rearrangements, including the formation of molecules possessing unusually long carbon–carbon single bonds. Fluorenyl-bearing propargylallenes react with silver nitrate or iron carbonyl to form novel organic polycyclic systems. Finally, suggestions are offered for future advances in the area.

## 1. Introduction

**Scheme 1:**
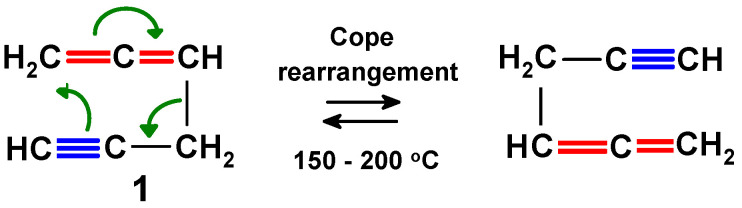
Cope rearrangement of propargylallene, **1**, showing interchange of the allene and alkyne functional groups [1].

While the published literature on organometallic derivatives of alkynes is enormous, the corresponding coverage of allenes is not yet at that level, but is still impressively large. Of course, these functional groups are frequently interconvertible, and a prime example is the [3,3]-sigmatropic shift (Cope rearrangement) of 1,2-hexadien-5-yne (propargylallene), **1**, into itself, as depicted in Scheme 1.

Alkynylallenes, with their potential to offer the varied reactivity patterns of two different multiple bond linkages, have attracted considerable attention, in particular, the ability of their metal complexes to undergo spectacular rearrangements. Initially, we discuss rather briefly the already comprehensively reviewed organometallic chemistry of alkynylallenes, whereby the unsaturated units are linked via tethers of different length, and list just a few typical examples of the many that have been reported. We then focus in greater detail on the more recent work on the syntheses and reactivity of derivatives of 1,2-hexadien-5-yne (propargylallenes) in which the allene and alkyne linkages are connected by only a single carbon atom bridge.

## 2. Thermal Cycloadditions and Rearrangements of Alkynylallenes

**Scheme 2:**
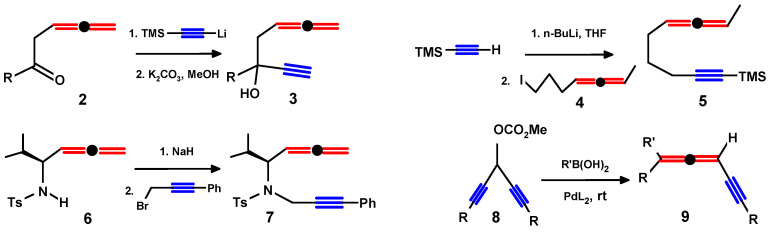
Examples of standard synthetic routes to alkynylallenes.

In recent years, intramolecular thermal reactions between alkyne and allene fragments have been extensively studied and excellent comprehensive reviews have appeared [2,3]. As exemplified in Scheme 2, the connecting chain between the functional groups may be varied in length and in chemical identity such that heteroatoms are frequently incorporated [4,5,6]. Typical synthetic routes involve either nucleophilic attack by a lithio-alkyne on an allenyl ketone, as in **2** → **3**, or on an iodoalkyl-allene **4** → **5**, or by reaction of an anion on a propargyl halide **6** → **7**. Very recently, it has been reported that the palladium-catalysed coupling of 1,4-diyn-3-yl carbonates, **8**, with boronic acids delivers allenynes, **9**, in excellent yields while tolerating a wide range of substituents [7].

**Scheme 3:**
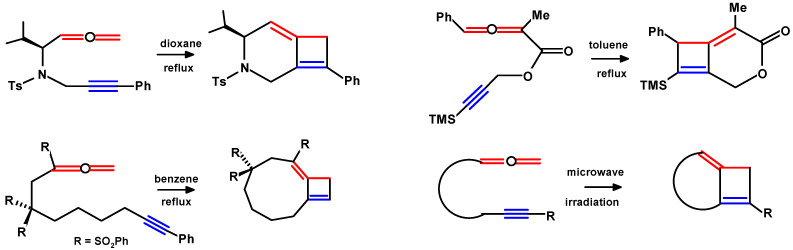
Selected examples of thermally initiated intramolecular cycloadditions of alkynes to allenes.

While rearrangements are generally initiated thermally, it has also been shown that microwave irradiation is another useful methodology. This approach led to shorter reaction times and enhanced yields. Examples of this approach are shown in Scheme 3, and include the preparation of bicyclo[4.2.0]octadienes and bicyclo[5.2.0]nonadienes [8,9].

**Scheme 4:**
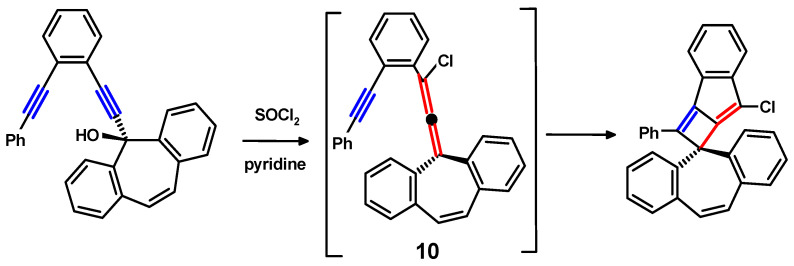
Intramolecular cyclisation via an allene intermediate.

In the example shown in Scheme 4, an initial Meyer–Schuster rearrangement step is required to generate the intermediate alkynyl-chloroallene, **10**, which then undergoes cyclisation to form the cyclobutene component [10].

**Scheme 5:**
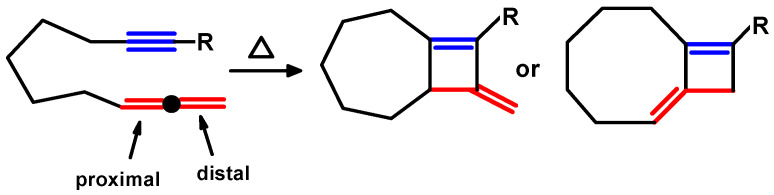
Cycloaddition can occur at either the proximal or distal allene double bond.

We note that, depending on the identity of the substituents, the [2+2] cycloaddition process can yield regioisomers, whereby the alkyne reacts with either the proximal or distal double bond of the allene, as illustrated in Scheme 5.

**Scheme 6:**
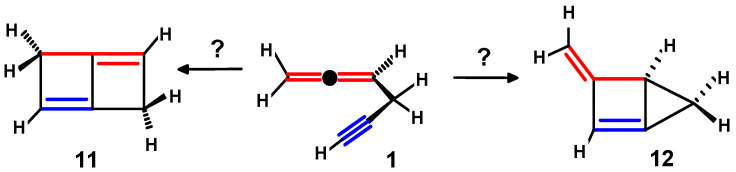
Isomers theoretically derivable, but experimentally not viable, via intramolecular cycloadditions of propargylallene, **1**.

However, in propargylallene, **1**, either of these cycloadditions is precluded because the shortness of the one-carbon linker between the allene and alkyne units would lead to highly strained systems. One can envisage two products of interaction between the two unsaturated moieties: *iso*-Dewar benzene, **11**, would result from [2+2] cycloaddition of the alkyne and the terminal (distal) double bond of the allene, and the other alternative would be the methylene-bicyclo[2.1.0]pentene, **12** (Scheme 6). Since these isomers of C_6_H_6_ lie ~140 kJ mol^−1^ higher than the enthalpy of propargylallene itself [11], their formation is extremely unlikely.

## 3. Metal-Mediated Cyclisations of 1,2-Dien-*n*-ynes

In recent years, inter- and intramolecular carbon–carbon coupling processes mediated by metals such as silver, gold or platinum have become a major focus in organic/organometallic chemistry. Indeed, entire issues of *Chemical Reviews* (in 2008 and in 2021) and of *Beilstein Journal of Organic Chemistry* (in 2011) appeared, including contributions from many of the leading workers in the field [12,13,14,15,16,17,18,19]. Moreover, a number of other reviews have been devoted to this important theme, in particular, concerning cyclisations of alkynylallenes that have been effected by treatment with silver, gold, rhodium, palladium or platinum salts [20,21,22,23,24,25,26,27,28,29,30], and also the use of other transition metals such as cobalt [31] or titanium [32].

**Scheme 7:**
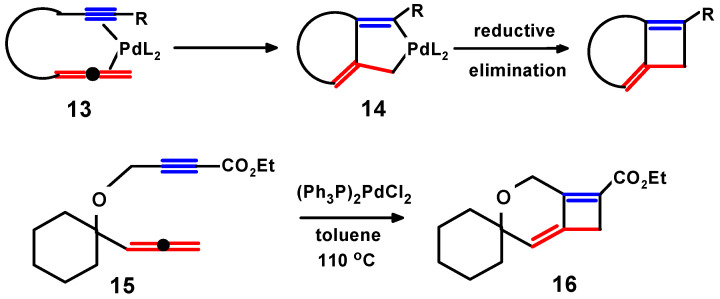
Palladium-catalysed intramolecular coupling of an alkyne and an allene.

While a range of mechanisms has been elucidated, a typical reaction mode is illustrated below. Palladium-catalysed cyclisations are thought to proceed by initial π-coordination to both the double bond and the triple bond in the substrate, as in **13**, followed by migratory C–C coupling to form a palladocyclopentene, **14**; the final step leading to product formation is reductive elimination, thereby releasing the palladium to be available for the next catalytic cycle [2,33]. A classic example of such a process, the conversion of **15** to **16**, is shown in Scheme 7 [8].

**Scheme 8:**
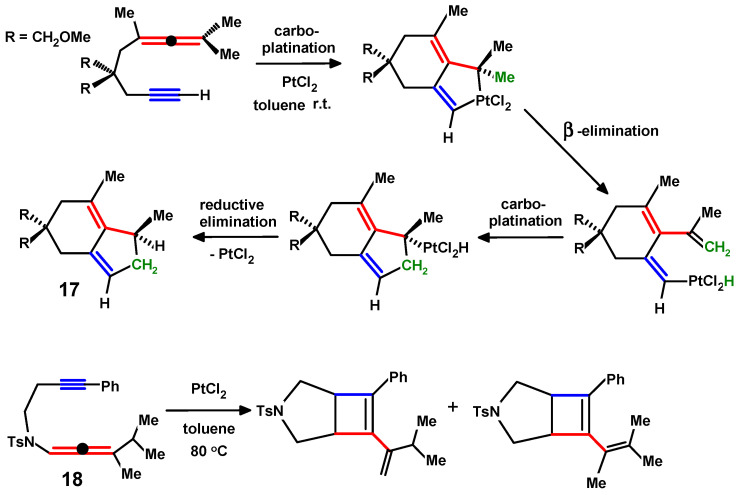
Platinum-mediated cycloisomerisation of alkynylallenes.

Impressively, platinum dichloride can even bring about analogous reactions at room temperature. Taking a specific example, Malacria reported that a wide range of 1,2-dien-7-ynes readily cyclise, presumably via a platinacyclopentene intermediate, to yield bicyclic products such as **17** (Scheme 8) [24]. Subsequently, Murakami [25] described the cycloisomerisation of a closely related system, **18**, possessing a tosylamino unit in the tether.

**Scheme 9:**
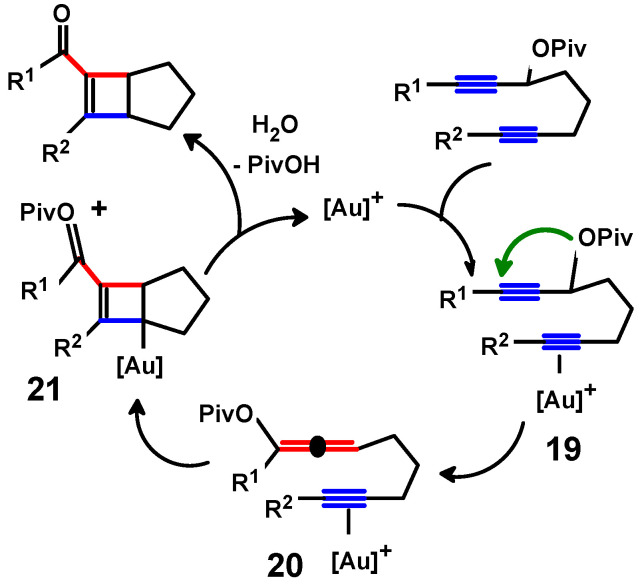
Proposed mechanism for the gold-catalysed coupling of a 1,2-dien-7-yne.

The use of Au(I) as a catalyst has been very widely studied and we show two cases as exemplars of the elegant work in this area. The transformation of bisalkynyl pivalates into tricyclic systems is shown in Scheme 9. The proposed mechanism involves the initial coordination of a gold(I) species to a triple bond, to yield the 1,7-diyne **19**, which then undergoes 1,3 migration of the pivalyl moiety to form the alkynylallene **20**. After [2+2] cycloaddition of the alkene and alkyne moieties, the gold is now σ-bonded, as in **21**, and, in the final step, loss of pivalate leads to product and regenerates the active gold catalyst [34].

**Scheme 10:**
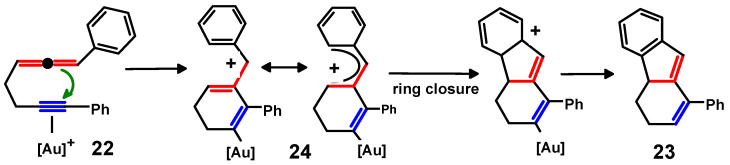
Gold(I)-mediated cyclisation of a 1,2-dien-6-yne to a dihydrofluorene.

Another fine example appears in Scheme 10 and involves the initial coordination of gold(I) to the alkyne, thereby bringing about cyclisation to the intermediate cyclohexadiene, **24**, now linked directly to a benzyl cation. Subsequent 4π electrocyclisation completes the sequence of consecutive ring closures from the 1,2-dien-6-yne **22** to the tricyclic system **23** [35].

The Pauson–Khand reaction (PKR), which entails the metal-catalysed [2+2+1] cycloaddition of an alkyne, an alkene, and CO, is one of the most widely used approaches towards the synthesis of cyclopentenones. Of particular relevance to the present discussion, we note that its intramolecular version leads to bicyclic products, whereby the size of the ring adjacent to the cyclopentenone is governed by the length of the tether connecting the alkene and alkyne units. However, when the alkene is replaced by an allene, either of the C=C double bonds of the allene may react preferentially with the alkyne moiety, thereby changing the size of the second ring.

**Scheme 11:**
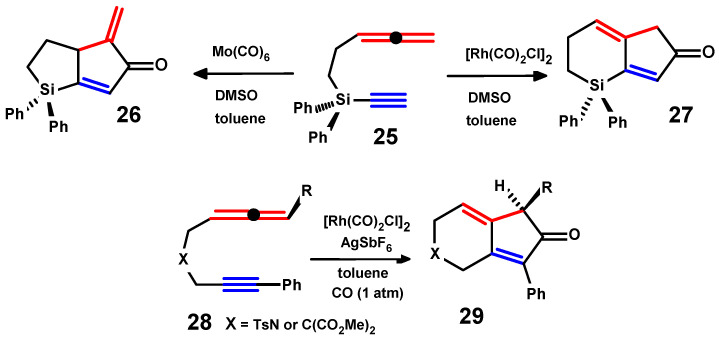
Pauson–Khand reactions catalysed by different metals.

As noted by Brummond, use of cobalt or molybdenum carbonyl as the catalyst for the PKR of the silicon-tethered 1,2-dien-7-yne **25** yields only the bicyclo [3.3.0] product **26**. In contrast, rhodium catalysis favours the formation of the isomeric bicyclo [4.3.0] framework **27**, i.e., reaction of the alkyne preferentially with the distal double bond of the allene [36]. More recently, this latter approach has been used to bring about efficient chirality transfer by taking advantage of the availability of axially chiral allenynes, as for **28** → **29**, shown in Scheme 11 [37,38].

**Scheme 12:**
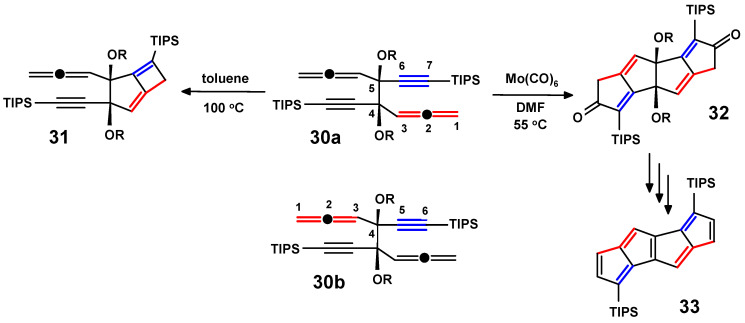
Cyclisation reactions of the bis(propargylallene), **30**.

It is noteworthy that the bis-propargylallene **30** can be viewed as possessing either a 1,2-dien-6-yne (**30a**) or a 1,2-dien-5-yne (**30b**) motif (Scheme 12). Thermolysis yields the bicyclo[3.2.0]heptadiene derivative **31** by [2+2] cycloaddition of the allene and alkyne moieties that are linked by the two-carbon bridge, as in **30a**, rather than a reaction between the propargylic allene and alkyne fragments depicted in **30b**. Moreover, **30** undergoes a tandem molybdenum–mediated Pauson–Khand reaction to provide the [5.5.5.5] tetracyclic diketone **32**; further manipulation allowed its conversion into the dicyclopenta[*a,e*]pentalene **33** [39].

**Scheme 13:**
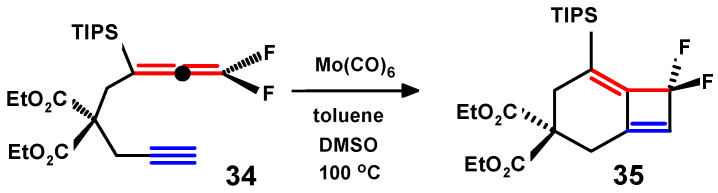
[2+2] Cycloaddition catalysed by Mo(CO)_6_.

Note, however, that markedly changing the electronic character of the substituents on the allene portion, as in the difluorinated example **34** [40], does not yield a PKR product but results only in [2+2] cycloaddition to form the bicyclo[4.2.0]octadiene **35** when allowed to react with Mo(CO)_6_ (Scheme 13).

## 4. Propargylallenes: Syntheses and Reactivity

### 4.1. Propargylallene: Structure and Spectroscopy

**Figure 1:**
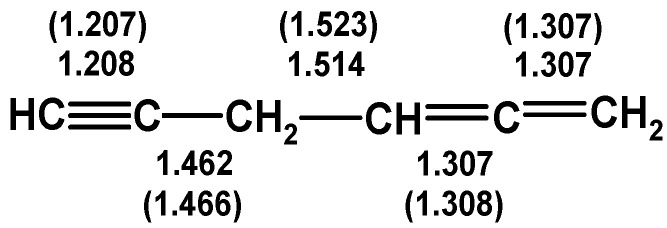
Experimental bond lengths (Å) in 1,2-hexadien-5-yne, **1** from electron diffraction data; DFT-calculated values in parentheses.

While the organic chemistry of molecules possessing the 1,2-dien-5-yne (propargylallene) motif has been comprehensively reviewed [41], their organometallic chemistry has been less well chronicled. The infrared spectrum of propargylallene itself exhibits ν(C≡C) and ν(C=C=C) stretching vibrations at 2124 and 1958 cm^−1^, respectively [42], and gives rise to a characteristic ^13^C NMR resonance at ~205 ppm for the central carbon C=***C***=C; its photoelectron spectrum has also been reported [43]. Since it is a liquid at ambient temperature, X-ray crystallographic data are not available, but its structure has been elucidated by electron diffraction in the gas phase [44]. Gratifyingly, the experimental bond lengths (Figure 1) are in excellent agreement with the values found from density functional calculations at both the B3LYP and MP2 levels of theory [11].

### 4.2. Syntheses of Propargylallenes

**Scheme 14:**
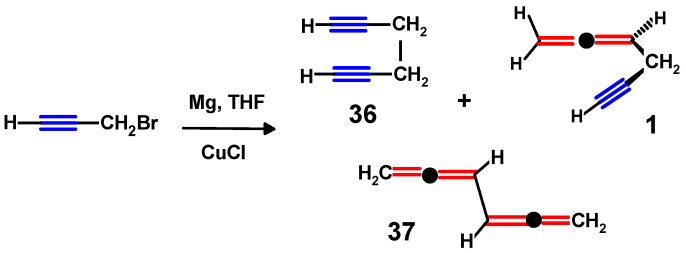
Copper-mediated coupling products derived from propargyl bromide.

Preparative routes to propargylallenes commonly lead to isomeric mixtures. Typically, as shown in Scheme 14, the reaction of propargyl bromide with magnesium and CuCl in THF leads to bipropargyl, **36**, biallenyl, **37**, and propargylallene **1**, whereby the composition of this product mixture varies and depends on the exact reaction and work-up conditions [45].

**Scheme 15:**
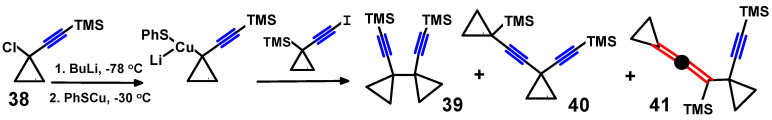
Copper-mediated coupling products derived from **38**.

Likewise, (Scheme 15) metallation of 1-chloro-1-(trimethylsilylethynyl)-cyclopropane, **38**, and then treatment with 1-iodoethynyl-1-trimethylsilylcyclopropane led to the 1,5-diyne **39**, the 1,4-diyne **40**, and the propargylallene **41** [46].

**Scheme 16:**
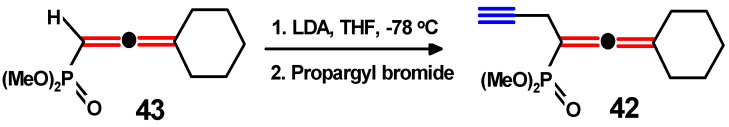
Synthesis of the propargylallenyl phosphonate **42**.

In contrast, (Scheme 16) the propargylallenyl phosphonate **42** was preparable in good yield by lithiation of the allenyl phosphonate **43** and reaction with propargyl bromide [47].

**Scheme 17:**
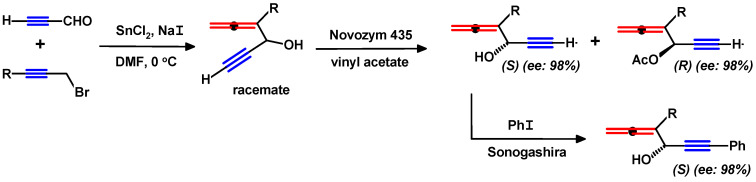
Enzyme-mediated preparation of optically pure propargyl–allenols.

Interestingly, it has been shown that Novozym-435 (a form of *Candida Antarctica lipase B*) can function as an effective biocatalyst, whereby a large number of optically pure 1-ethynyl-substituted 2,3-allenols were prepared by kinetic resolution of the initial racemic products. When carried out in vinyl acetate solution at 30 °C, this procedure afforded *(S)*-2,3-allenols and *(R)*-2,3-allenyl acetates in high yields and excellent *ee* values. The initial racemic materials were conveniently prepared by the reaction of the appropriately substituted propargyl bromide and propynal, when mediated by SnCl_2_ and NaI in DMF. Subsequently, it was reported that these enantiomerically pure propargyl–allenols underwent Sonogashira coupling reactions with excellent retention of stereochemical integrity (Scheme 17) [48].

**Scheme 18:**
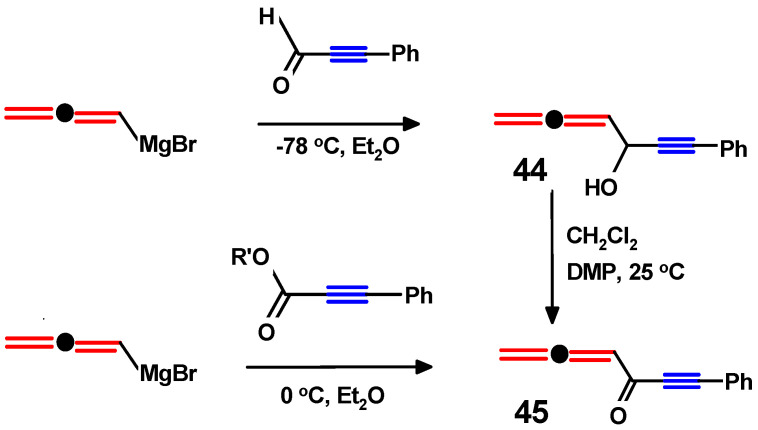
Synthetic routes to an alkynyl allenyl ketone.

Propargyl–allenols and ketones, such as **44** and **45**, respectively, are also preparable via the reaction of an allenyl Grignard reagent with the appropriate alkynal or alkynyl ester, as in Scheme 18 [49].

Turning now to the preparation of propargylallenes bearing aromatic substituents; whereas the monophenyl–propargyl alcohol, Me_3_Si-C≡C-CH(Ph)OH, **46**, reacted with a range of Lewis acids to generate the bis(phenylpropargyl) ether **47**, the behaviour of the corresponding diphenyl–propargyl alcohol, **48**, was found to be dependent on the identity of the Lewis acid [50].

**Scheme 19:**
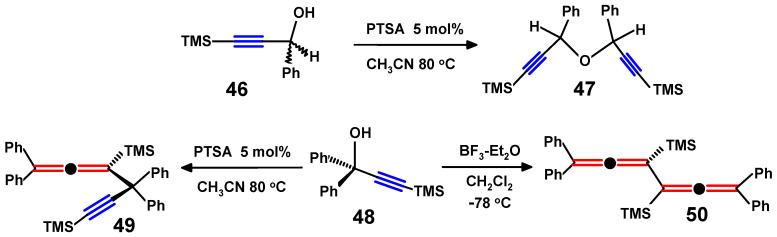
Reactions of the monophenyl–propargyl alcohol **46**, and the diphenyl–propargyl alcohol **48**, with different Lewis acids.

Thus, treatment of **48** with a catalytic quantity of p-toluenesulfonic acid (PTSA) led to 3,6-bis(trimethylsilyl)-1,1,4,4-tetraphenyl-1,2-hexadien-5-yne, **49**, which was characterised by X-ray crystallography. In contrast, as shown in Scheme 19, the corresponding reaction with BF_3_·Et_2_O led to the diallene **50**, the result of a tail-to-tail reductive dimerisation.

**Scheme 20:**
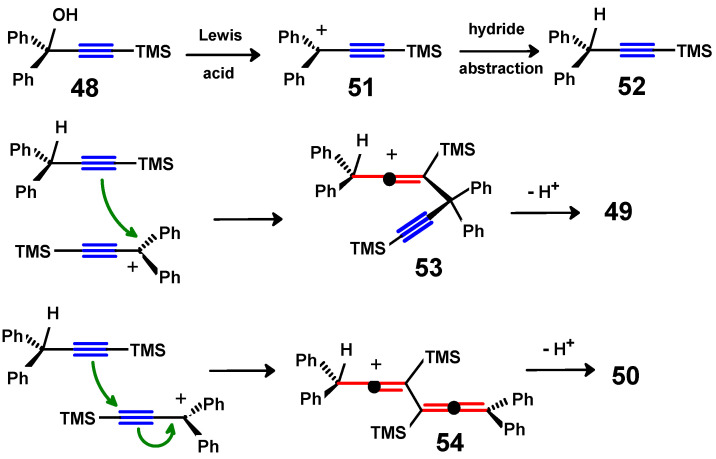
Proposed mechanisms for the formation of allenyne, **49**, and diallene, **50**.

These observations were rationalised by Chauvin (Scheme 20) in terms of the initial production of the pseudo-trityl cation **51**, which abstracts a hydride to form **52**. Subsequent nucleophilic attack by **52** on **51** can occur either at the cationic site, to generate **53**, or at the silylated terminal of the triple bond, thus forming **54**; loss of a proton delivers the observed allenyne **49** and the diallene **50**, respectively [50].

### 4.3. Di-Aroyl-Propargylallenes and Diallenes

**Scheme 21:**
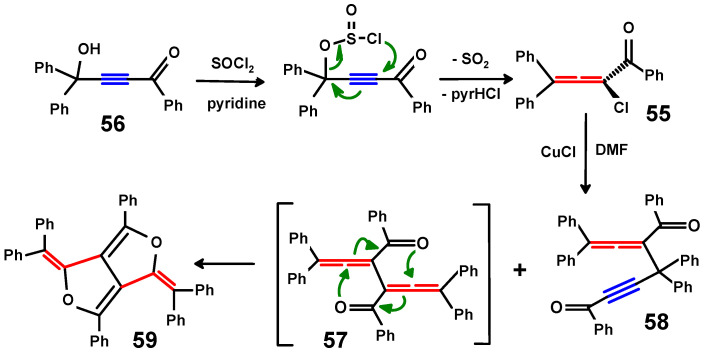
Attempted synthesis of the diallene, **57**, led instead to the propargylallene, **58**, and the furofuran, **59**.

When 1-benzoyl-1-chloro-3,3-diphenylallene, **55**, readily prepared by Meyer–Schuster rearrangement of the alkynol **56** by reaction with thionyl chloride, was subjected to copper chloride-promoted coupling with the aim of forming the diallene, **57**, two unexpected products were observed [51,52]. Instead, the isomeric propargylallene, 3,6-dibenzoyl-1,1,4,4-tetra-phenyl-1,2-hexadien-5-yne, **58**, arising from head-to-tail coupling, along with the furofuran, **59**, were isolated and characterised spectroscopically. Apparently, the desired tail-to-tail allene dimer, **57**, is not stable, even at room temperature, and undergoes a facile 8π-electron thermal cyclisation (Scheme 21).

**Scheme 22:**
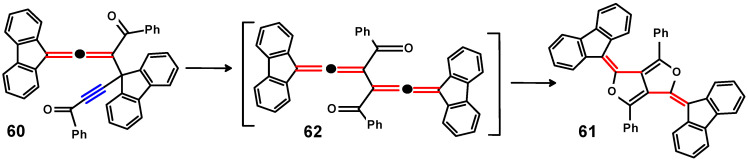
Cyclisation of the propargylallene, **60** → **61**, via the diallene **62**.

**Figure 2:**
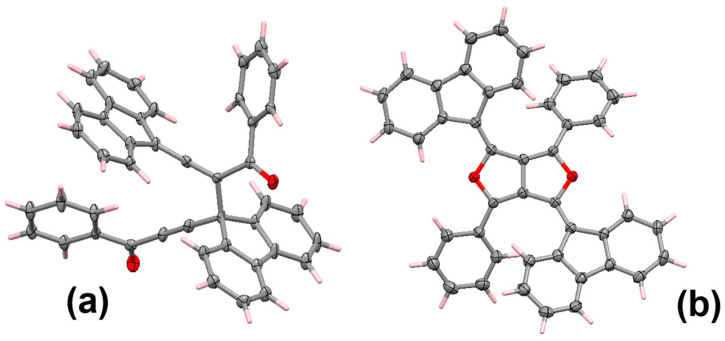
Molecular structures of (**a**) the propargylallene, **60**, and (**b**) the furofuran, **61**.

When the geminal phenyl groups in **56** were replaced by a fluorenyl substituent, the propargylallene **60**, formed upon treatment with CuCl, was readily isolable. Moreover, thermolysis of **60** furnished the corresponding furofuran **61**, presumably via the diallene **62** (Scheme 22 and Figure 2). This prompted a study of the reaction in the crystalline state in which it was shown that, even below the melting points of the reactants, the rearrangement of **60** → **61** proceeds readily [53,54]. When the reaction was carried out using unsymmetrically substituted propargylallenes, no cross-over products were observed, thereby verifying the intramolecular character of the rearrangement process.

### 4.4. Metal-Mediated Rearrangements of Propargylallenes

**Scheme 23:**
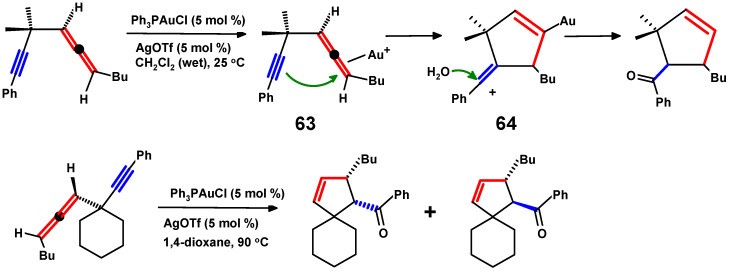
Gold-catalysed cyclisations with initial coordination at the allene.

Although there are many examples of 1,*n*-enynes that undergo metal-catalysed intramolecular cycloaddition, propargylallenes have been much less investigated, and one can envisage many opportunities for growth in this area. In particular, the identity of the products can vary dependent on whether initial coordination occurs at the alkyne or the allene functionality. As illustrated in Scheme 23, initial coordination of the metal to the outer double bond of the allene leads to five-membered ring formation, which, when followed by nucleophilic attack at the terminal carbon of the alkyne, leads to a ketone. In the two examples shown, coordination of a gold(I) species to the allenyl fragment, as in **63**, led to the formation of a five-membered ring, and subsequent attack by water at the exocyclic vinyl cation, as in **64**, ultimately yielded a ketone [55].

**Scheme 24:**
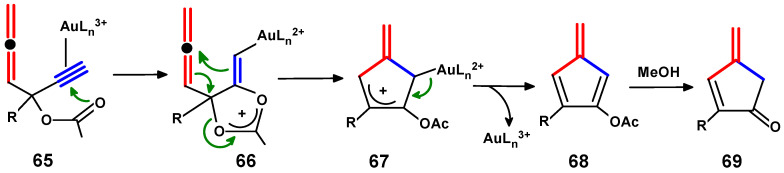
Gold(III)-catalysed rearrangement of propargylallenyl acetates into 4-methylene-2-cyclopentenones.

**Scheme 25:**
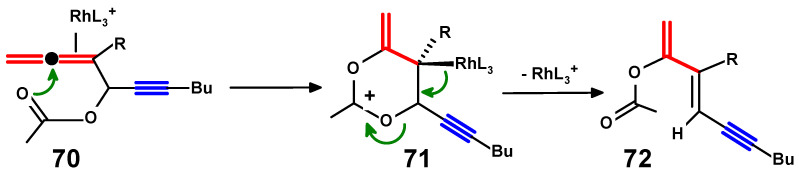
Rhodium-catalysed rearrangement of a propargylallenyl acetate.

The differing consequences of the initial coordination of the metal to either the alkyne or the allene by gold(III) or rhodium(I), respectively, are clearly exemplified in Scheme 24 and Scheme 25. In the first case, reaction with Au(III) brings about the conversion of propargylallenyl acetates into 4-methylene-2-cyclopentenones. This rearrangement is thought to proceed by the initial coordination of gold to the alkyne unit, as in **65**, thus rendering it susceptible to nucleophilic attack by the carbonyl oxygen of the acetate, thereby forming a vinyl–gold intermediate **66**. Subsequent cyclisation produces the cationic intermediate **67,** which, after elimination of the gold(III) unit, delivers the fulvene **68**; finally, methanolysis yields the final product **69** [56].

In the alternative scenario, rhodium-catalysed 1,3-acetoxyl rearrangement of propargylallenyl acetates leads to *E*-diene-dynes [57]. In this case, Rh(I) coordinates initially to the internal double bond of the allenyl fragment in **70,** thereby bringing about attack by the carbonyl oxygen of the ester to form the cyclic cation **71**; finally, loss of the catalytic rhodium species results in the migration of the acetate and formation of the *E*-diene-yne, **72** (Scheme 24).

## 5. Propargylallenes and the Ruthenium Route to *gem*-Dialkynylmethanes

**Scheme 26:**
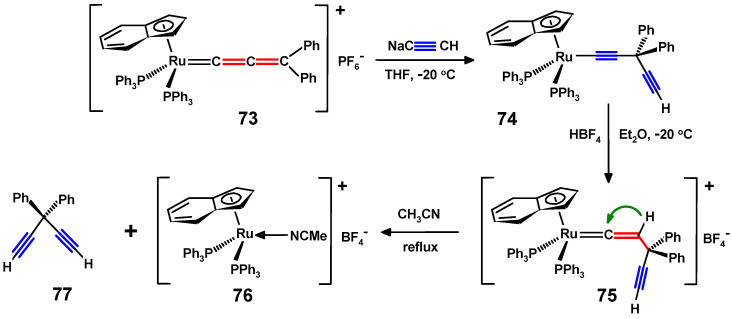
Synthetic route to diphenyl-dipropargylmethane.

The search for a convenient synthetic route to *gem*-dialkynylmethane derivatives was finally resolved by Chauvin. This long-sought target was finally obtained via the nucleophilic attack of sodium acetylide on the cationic ruthenium–allenylidene complex **73**, thereby forming the diyne **74**, bearing the organo-ruthenium moiety (Scheme 26). Protonation of **74** delivered the vinylidene derivative, **75**, which, upon heating at reflux in acetonitrile, released diphenyl-dipropargylmethane, **77**, along with the cationic ruthenium complex **76** [50].

**Scheme 27:**
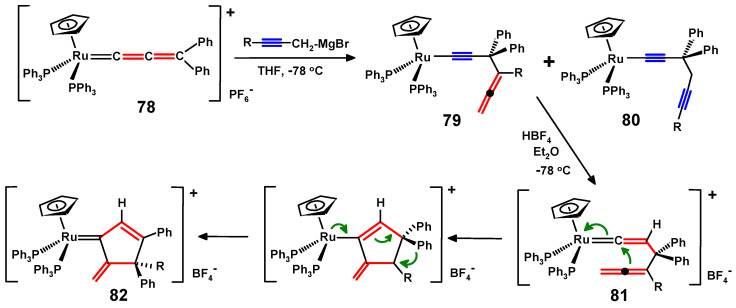
Cyclisation of a ruthenium propargylallene upon protonation.

This chemistry was extended by treatment of the ruthenium allenylidene cation **78** with the propargyl Grignard reagents RC≡C-CH_2_MgBr (R = Me, Et, Ph), which yielded the propargylallenes, **79**, as major products (90%), and diynes, **80**, as the minor ones. Protonation of the former with HBF_4_ resulted in cyclisation, via a vinylidene intermediate **81**, with subsequent phenyl migration to form the methylene–cyclopentenylidene complexes **82**, as shown in Scheme 27.

**Scheme 28:**
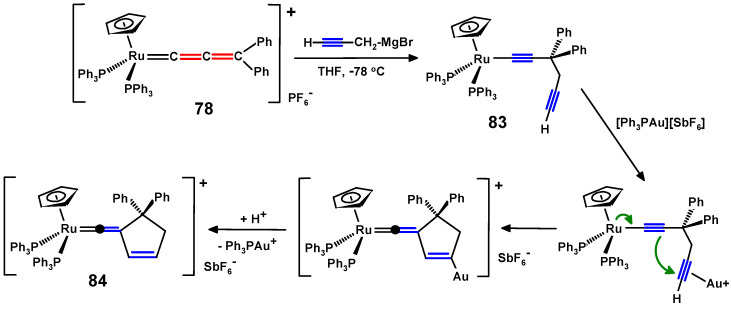
Cyclisation of a ruthenium 1,5-diyne upon treatment with gold(I).

In contrast, the reaction of the ruthenium cation, **78**, with the parent propargyl Grignard reagent, HC≡C-CH_2_MgBr, led only to the diyne, **83**, that underwent a different cyclisation process when treated with [Ph_3_PAu][SbF_6_]. As depicted in Scheme 28, coordination of the gold(I) species to the terminal alkyne induced ring closure to the ruthenium–vinylidene cation, **84** [58].

## 6. Propargylallenes from Alkynylfluorenols

### 6.1. From Allenes to Tetracenes

**Scheme 29:**
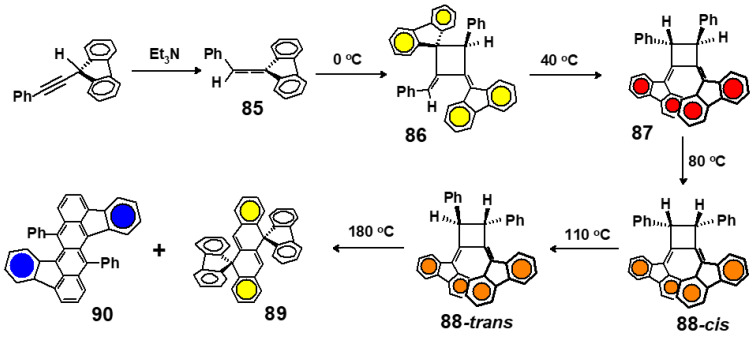
Synthetic route to the tetracenes **89** and **90**.

**Figure 3:**
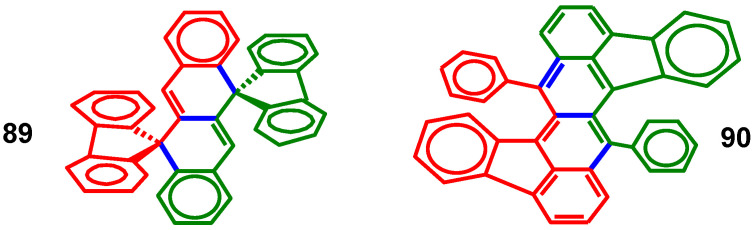
Positioning of the original 9-phenylethynylfluorene units in tetracenes **89** and **90**.

As part of a programme towards the syntheses of electroluminescent tetracenes, the base-mediated rearrangement of 9-phenylethynyl-[9*H*]fluorene into 3,3-(biphenyl-2,2′-diyl)-1-phenylallene, **85**, followed by head-to-tail dimerisation to form yellow 3-(9-fluorenyl-idene)-2-phenyl-4-(phenylmethylene)spiro[cyclobutane-1,9′[9*H*]fluorene], **86**, and thermal rearrangement to yield the red dimer **87**, then led consecutively to the orange *cis* and *trans* isomers of the tail-to-tail dimer 3,4-diphenyl-1,2-bis(fluorenylidene)cyclobutane, **88**, and ultimately the yellow dispirotetracene **89**, and the blue di-indenotetracene **90** (Scheme 29) [59,60,61]. Each of these intermediate steps was characterised by X-ray crystallography, and the two different alignments of the original allene motifs in the tetracene skeleton are illustrated in Figure 3.

### 6.2. Bromo and Silyl Derivatives of Fluorenylideneallenes

**Scheme 30:**
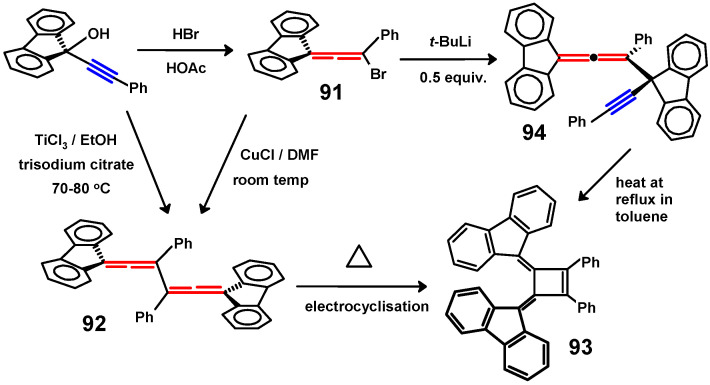
Successive formation of bromoallene **91**, diallene **92**, cyclobutene **93** and propargylallene **94**.

**Figure 4:**
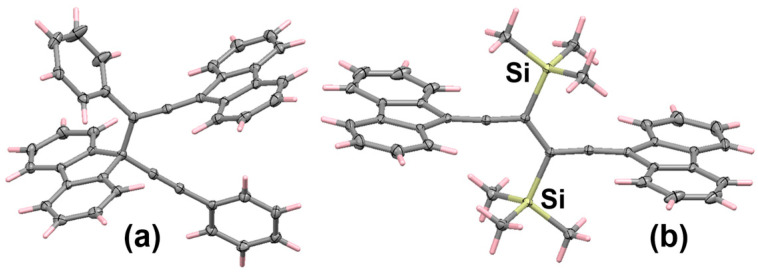
Molecular structures of (**a**) 3,3-(biphenyl-2,2′-diyl)-1-phenyl-1-[9-phenylethynyl-9*H*-fluorenyl]allene, **94**, and (**b**) of *s-trans*-1,6-bis((biphenyl-2,2′-diyl)-3,4-bis(trimethylsilyl)-1,2,4,5-hexatetraene, **97**.

When 3,3-(biphenyl-2,2′-diyl)-1-bromo-1-phenylallene, **91**, prepared by the reaction of 9-phenylethynyl-[9*H*]fluoren-9-ol with hydrogen bromide in acetic acid, was allowed to react with copper(I) chloride at room temperature, it yielded the diallene **92,** which, upon thermolysis, underwent cyclisation to the cyclobutene **93** (Scheme 30). In contrast, treatment of the bromoallene **91** with half an equivalent of butyllithium delivered the diphenyl-propargylallene, **94**, as pale-yellow crystals, in 43% yield; its X-ray crystal structure (Figure 4) reveals allene double-bond distances of 1.303 and 1.315 Å, and an alkyne triple bond of 1.198 Å [62,63].

**Scheme 31:**
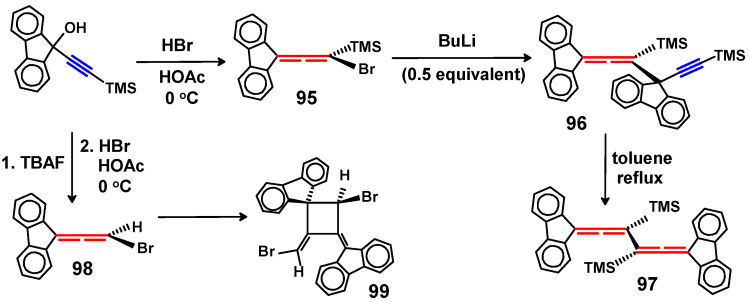
Preparative routes to the disilyl-propargylallene **96**, and the disilyl-diallene **97**.

Likewise, as shown in Scheme 31, treatment of 3,3-(biphenyl-2,2′-diyl)-1-bromo- 1-(trimethylsilyl)allene, **95**, with only half an equivalent of butyllithium, yielded 3,3-(biphenyl-2,2′-diyl)-1-(trimethylsilyl)-1-[9-trimethylsilylethynyl)-9*H*-fluorenylallene, **96**, which upon heating at reflux in toluene isomerised to form the diallene **97** that adopted the *s-trans* conformation with allene double-bond distances of 1.318 and 1.306 Å [64,65]; the molecular structure of **97** is also shown in Figure 4.

**Figure 5:**
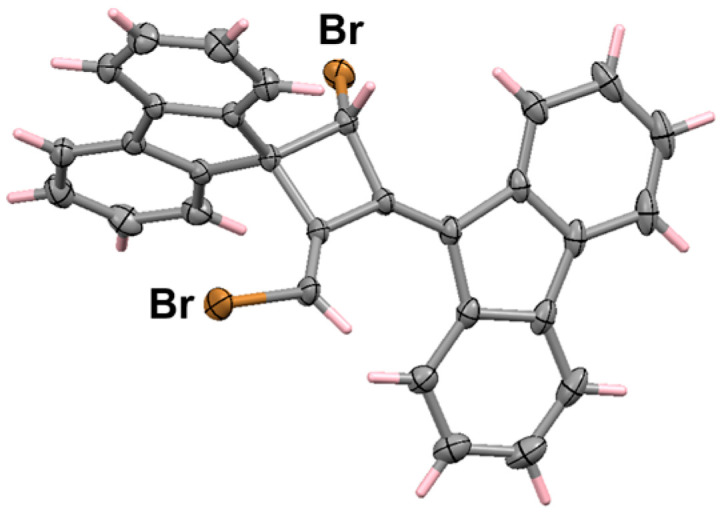
Molecular structure of 3-(9-fluorenylidene)-2-bromo-4-(bromomethylene)spiro-[cyclobutane-1,9′-[9*H*]fluorene], **99**.

It is also noteworthy that, when 9-trimethylsilylethynyl-[9*H*]fluorenol was desilylated using TBAF and the product allowed to react with HBr, the resulting 3,3-(biphenyl-2,2′-diyl)-1-bromoallene, **98**, underwent ready head-to-tail dimerisation to form the cyclobutene **99** (Scheme 31), whose structure appears as Figure 5 [64].

### 6.3. Reactions of Fluorenylidene Propargylallenes with Silver Nitrate

**Scheme 32:**
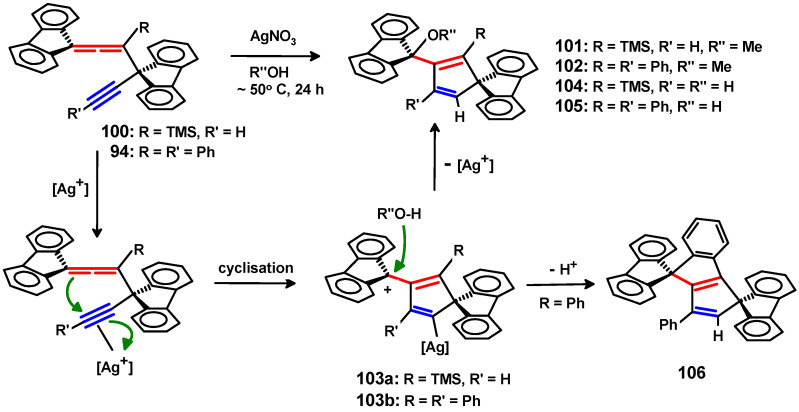
Silver-promoted cyclisation of propargylallenes with initial coordination at the alkyne.

**Figure 6:**
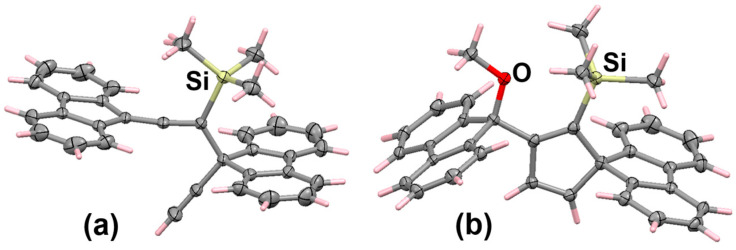
Molecular structures of (**a**) the monosilylpropargylallene **100**, and (**b**) the cyclopentadiene **101**.

As noted earlier, treatment of alkynylallenes with coinage metals frequently brings about molecular rearrangements. To this end, when a solution of the bis(trimethylsilyl)propargylallene, **96**, in methanol/water was stirred at room temperature with AgNO_3_, the terminal TMS group was eliminated to yield 3,3-(biphenyl-2,2′-diyl)-1-(trimethylsilyl)-1-ethynyl)-[9*H*]fluorenylallene, **100**. However, when either the monosilylpropargylallene, **100**, or the diphenyl-propargylallene, **94**, was heated at ~50 °C for 24 h in methanol together with silver nitrate, coupling of the allene and the alkyne led to the formation of the cyclopentadienes **101** or **102**, respectively; in each case, the newly formed ring was spiro-linked to a 9-methoxy-[9*H*]-fluorenyl substituent (see Figure 6). The mechanistic rationale invoked the initial coordination of Ag^+^ to the alkyne, prompting cyclisation to form the cationic five-membered ring system, **103**, that suffered nucleophilic attack by methanol to yield the final product (Scheme 32).

**Figure 7:**
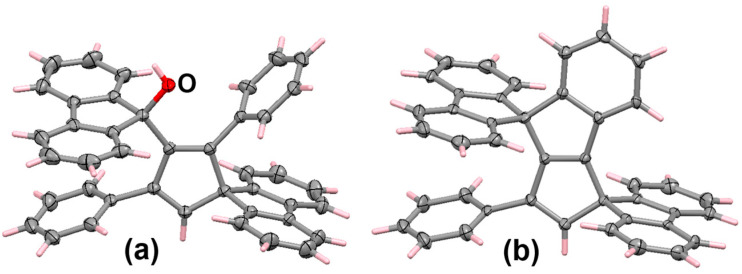
Molecular structures of (**a**) the cyclopentadiene **104** and (**b**) the dihydrobenzpentalene, **106**.

The reactions of propargylallenes **100** and **94** with AgNO_3_ in aqueous acetone, to give **104** and **105**, respectively, proceed in an analogous fashion to form products now containing a 9-hydroxy-9*H*-fluorenyl group bonded to the cyclopentadiene, again bearing a spiro-bonded fluorene. We note, however, that in the diphenyl system, when the intermediate cation, **103b**, was quenched with water, rather than methanol, the alcohol **105** (Figure 7) was now only a minor product; instead, the major product is the dispirofluorenyl-dihydrobenzpentalene, **106**, arising from Friedel–Crafts alkylation of the adjacent phenyl ring [63].

### 6.4. Ferrocenyl Derivatives of Propargylallenes

The synthesis of “push-pull” allenes, whereby one of the terminal substituents is electron-donating and the other electron-accepting, and the central carbon has carbenic character, has been the subject of a number of reports [66,67,68]. To this end, since it is well established that the ferrocenyl substituent is particularly effective at stabilising an adjacent carbocation, whereby charge is delocalised onto the iron [69,70], a series of ferrocenyl-containing allenes was prepared.

**Scheme 33:**
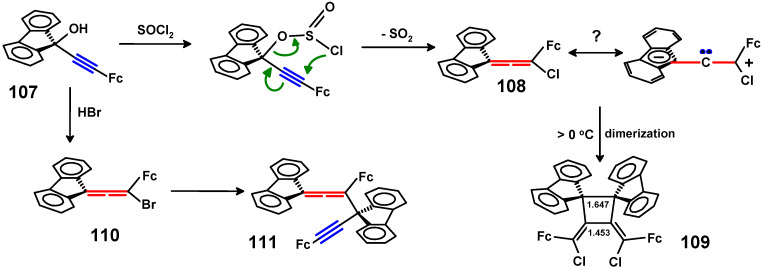
Reactivity of the ferrocenyl-haloallenes, **108** and **110**.

**Figure 8:**
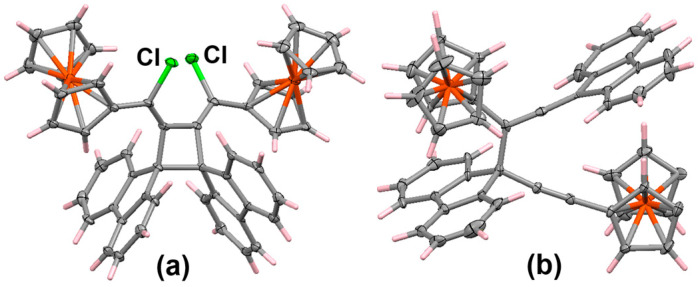
Molecular structures of (**a**) bis[(chloro)ferrocenylmethylene]-dispiro-fluorenylcyclo- butane, **109**, and (**b**) the diferrocenylpropargylallene, **111**.

When 9-ferrocenylethynyl-9*H*-fluoren-9-ol, **107**, was treated with thionyl chloride in the expectation of obtaining 3,3-(biphenyl-2,2′-diyl)-1-chloro-1-ferrocenylallene, **108**, via elimination of SO_2_ and HCl from the intermediate chlorosulfite (Scheme 33), the target molecule was not isolable and could only be detected spectroscopically. However, when the chloroallene, **108**, was allowed to warm gradually to room temperature, the product was identified as 1,2-bis[chloro(ferrocenyl)methylene)]-3,4-di(spiro-fluorenyl)-cyclobutane, **109**. X-ray crystallography revealed that the four-membered ring in this head-to-head dimer adopts a marked butterfly conformation in response to the pronounced twisting of the double bonds that minimises the steric crowding of the two vinylic chlorines [71]. However, the most striking feature of the structure shown in Figure 8 is the very long (1.647 Å) bond [72] connecting the spiro positions of the fluorenyl groups, which are themselves severely arced away from each other.

In contrast, when 9-ferrocenylethynyl-9*H*-fluoren-9-ol, **107**, was treated with cold aqueous HBr in the expectation of obtaining 3,3-(biphenyl-2,2′-diyl)-1-bromo-1-ferrocenylallene, **110**, a different product was obtained, and identified X-ray crystallographically as the head-to-tail dimer 1,1,4,4-bis(biphenyl-2,2′-diyl)-3,6-diferrocenyl-1,2-hexadien-5-yne, **111** (Figure 8) [71]. This product presumably arose from radical cleavage of the allenyl bromide linkage. We are unaware of any previously reported ferrocenyl-propargylallenes, although ferrocenyl derivatives of diynes, vinylallenes and diallenes have been reported [73].

### 6.5. Reactions of Fluorenylideneallenes with Iron and Cobalt Carbonyls

**Scheme 34:**
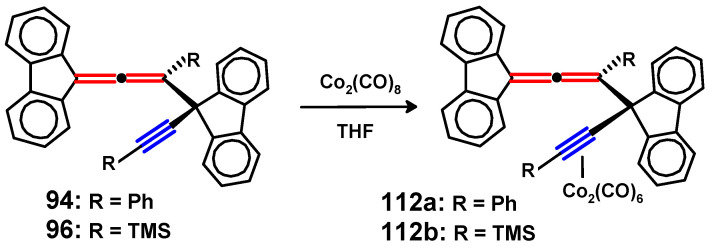
Reaction of propargylallenes **94** and **96** with dicobalt octacarbonyl.

The reactions of the diphenyl- and bis(trimethylsilyl)-propargylallenes, **94** and **96**, respectively, with dicobalt octacarbonyl were investigated to study the possibility of their undergoing Pauson–Khand coupling of the allene and the alkyne. It was also thought that, instead of forming the corresponding cyclopentenone, the reaction might stop at an intermediate step, thereby providing further information on the detailed mechanism of the PKR process [74,75,76]. Nevertheless, as shown in Scheme 34, in each case, the only product was the tetrahedral alkyne-Co_2_(CO)_6_ cluster **112** [63].

**Scheme 35:**
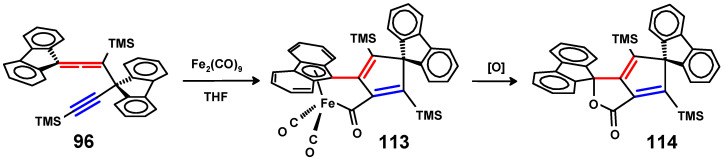
Reaction of the disilyl-propargylallene, **96**, with Fe_2_(CO)_9_, to form **113**, and its subsequent oxidative decomposition to form the lactone **114**.

These observations contrast with the behaviour of propargylallenes **94** and **96** with di-iron nonacarbonyl that yield organometallic products, whereby the alkyne and allene fragments are linked in a different fashion in the two cases. As shown in Scheme 35, the bis(trimethylsilyl)propargylallene, **96**, reacts with Fe_2_(CO)_9_ in THF to form a novel complex, **113**, in which an (η^5^-fluorenyl)Fe(CO)_2_ moiety is linked both directly and via a bridging carbonyl to a cyclopentadiene ring possessing two trimethylsilyl groups and a spiro-bonded fluorenyl substituent. Moreover, when left for several hours at room temperature in chloroform, this fluorenyl–iron complex suffered oxidative loss of the metal to yield an organic compound, **114**, whose structure was established by X-ray crystallography. The resulting bicyclic lactone is clearly derived from **113** by loss of the dicarbonyliron fragment and incorporation of an additional oxygen [63].

**Scheme 36:**
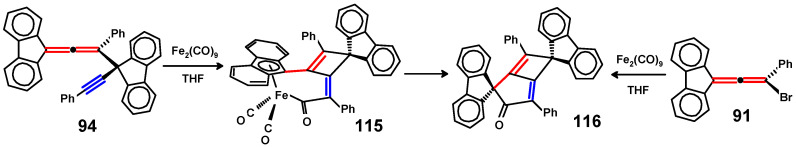
Reaction of the diphenyl-propargylallene, **94**, with Fe_2_(CO)_9_, to form **115**, and its subsequent decomposition to form the bicyclo[3.2.0]heptadienone, **116**.

In contrast, the product of the corresponding reaction of the diphenyl-propargylallene, **94**, with Fe_2_(CO)_9_ (or better with Fe(CO)_5_ and morpholine *N*-oxide) in each case exhibited ^1^H and ^13^C NMR spectra, indicating the presence of two different fluorenyl groups and two non-equivalent phenyl substituents. The product, **115**, was unambiguously identified by X-ray crystallography, which revealed that, as in **113**, it possesses an (η^5^-fluorenyl)iron-dicarbonyl moiety, and also a second fluorenyl substituent but now spiro-bonded instead to a cyclobutene (Scheme 36). This four-membered ring is bonded directly to C(9) of the complexed fluorenyl system, and is also attached to the iron atom via a =C(Ph)-C(=O)- linkage. This complex also suffers reductive elimination to yield an organic product shown by X-ray crystallography to be the bicyclo[3.2.0]heptadienone, **116**, that was also preparable directly from Fe_2_(CO)_9_ and the phenyl-bromoallene, **91**.

**Scheme 37:**
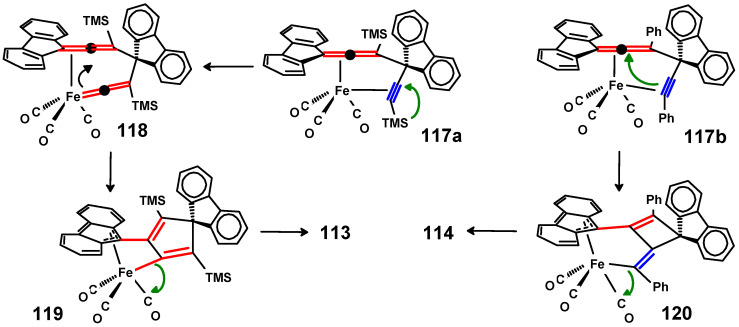
Mechanisms of reactions of propargylallenes with iron carbonyl.

The mechanistic rationale presented in Scheme 37 invokes the initial coordination of an Fe(CO)_3_ fragment to both the alkyne and allene units to be followed either (i) by rearrangement of the (propargylallene)Fe(CO)_3_, **117a** (R = TMS), into a silyl–vinylidene complex, **118**, that underwent cyclisation to **119**, and then to **113**, or (ii) in the phenyl case, **117b**, by direct rearrangement to the cyclobutene, **120**; subsequent migration onto a terminal carbonyl group yields the observed product, **115**. Evidently, the formation of the cyclopentadiene or cyclobutene ring depends on whether the alkynyl substituent in the primary intermediate, **117**, can migrate (yes for TMS, no for Ph) to form the appropriate vinylidene intermediate [63].

## 7. Suggestions for Future Developments

**Scheme 38:**
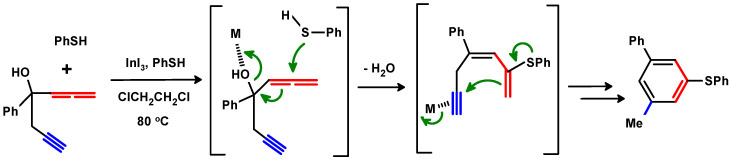
InI**_3_**-catalysed reaction of a hydroxylated allenyne with thiophenol.

While the use of cationic gold or silver complexes to bring about rearrangements of alkynylallenes is well established, we note a subsequent report whereby indium or zinc iodides, in the presence of thiophenol, initiate the conversion of alkynylallenols into aromatics bearing a thiophenyl substituent (Scheme 38) [77]. Evidently, a wide range of Lewis acids can mediate such chemistry.

**Scheme 39:**
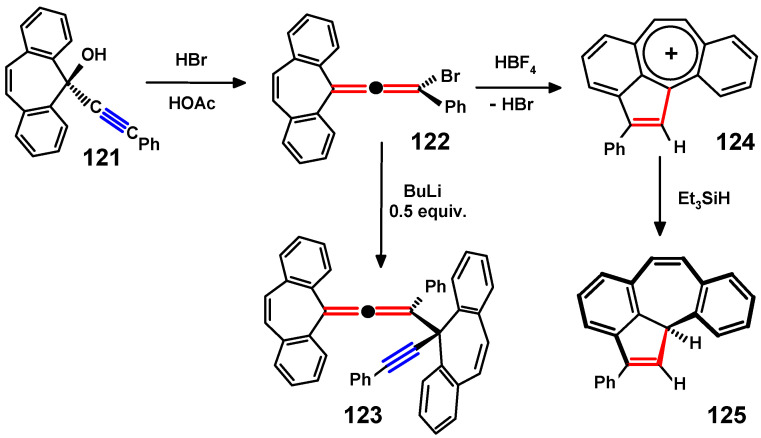
Reactions of 1-bromo-1-phenyl-2-dibenzo[*a,d*]cycloheptenylidene-ethene, **122**.

The versatile chemistry of fluorenylideneallenes and their derived propargylallenes prompted investigation of their dibenzosuberenylidene analogues. This system differs from fluorenyl in that it stabilises aromatic cations rather than anions; moreover, when uncharged, as in the alkynol, **121**, the central seven-membered ring adopts a boat conformation. As with the fluorenyl analogue, **91**, treatment of the bromoallene **122** with butyllithium furnished the propargylallene **123** (Scheme 39). However, as far as we are aware, its reactivity towards gold or silver cations or metal carbonyls has not been investigated, and we await with interest any future reports. In this vein, the dibenzoylpropargylallenes, **58** and **60** (Scheme 21 and Scheme 22), as well as their derived furofurans, **59** and **61**, merit investigation of their organometallic chemistry.

**Figure 9:**
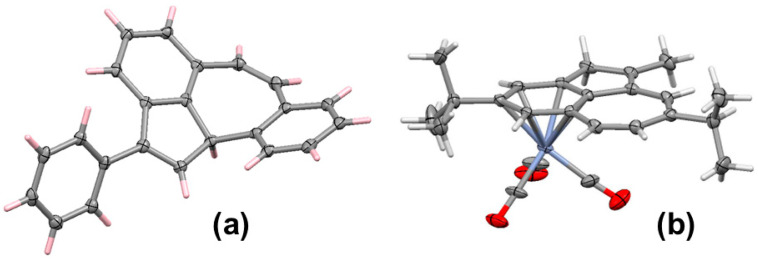
Molecular structures of (**a**) 2-phenyl-11b*H*-dibenz[*cd,h*]azulene, **125**, and (**b**) (η^6^-1-methyl- 8-isopropyl-4-*tert*-butylbenz[*cd*]azulene)CrCO)_3_, **126**.

Interestingly, as shown in Scheme 39, protonation of the central carbon of the bromoallene, **122**, followed by loss of HBr, yielded the tetracyclic tropylium cation, **124**, which, when reduced with triethylsilane, furnished 2-phenyl-11b*H*- dibenz[*cd,h*]azulene, **125**; X-ray crystallography revealed its bowl-shaped structure, as depicted in Figure 9 [78]. The chemistry of benzazulenes also bears further examination in light of their possible molecular rearrangements via η^5^ ↔ η^6^ ↔ η^7^ haptotropic shifts of organometallic fragments across the polycyclic skeleton, as in the complex, **126**, shown in Figure 9 [79].

**Scheme 40:**
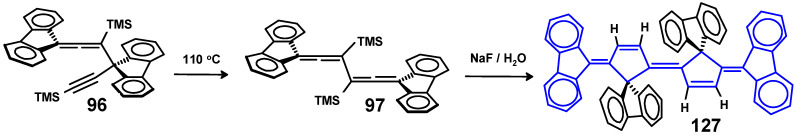
Cyclisation of the diallene, **97**, to form the *quater*-cyclopentadiene, **127**, in which the C_36_ component is clearly indicated.

Finally, we note the unexpected result of treating *s-trans*-1,6-bis((biphenyl-2,2′-diyl)- 3,4-bis(trimethylsilyl)-1,2,4,5-hexatetraene, **97**, with potassium fluoride to remove the silyl groups that led instead to the *quater*-cyclopentadiene, **127**, (Scheme 40), whose octacyclic C_36_ framework represents 60% of C_60_ and can be mapped directly onto the parent fullerene [65].

## 8. Conclusions

Propargylallenes offer the varied reactivity patterns of two different multiple bond linkages either separately or in concert. Their syntheses, structures, rearrangement mechanisms and synthetic utility, especially when treated with transition metal reagents such as gold(I), silver(I), platinum metals or metal carbonyls, reveal a wealth of novel and exciting chemical behaviour. One can clearly see how the juxtaposition of allenyl and alkynyl moieties, as found in propargylallenes, expediates metal-mediated interactions between the unsaturated groups. Coordination of metals such as silver, gold, platinum or rhodium to either the alkyne or the allene fragment can lead to a wide range of cyclisation or rearrangement products.

The successful isolation and characterisation of organometallic systems, such as **113** and **115**, in which cyclisation has already occurred, but the organometallic moiety is still coordinated, is in accord with established mechanistic proposals for the metal-mediated couplings in 1,*n*-ene-ynes. In the particular cases **113** and **115**, in which the newly formed ring sizes differ, the formation of complexes possessing cyclopentadiene or cyclobutene rings is controlled by the migratory aptitude of the alkynyl substituent in the initial Fe(CO)_3_ complex, whereby the trimethylsilyl, but not the phenyl, undergoes facile rearrangement to form a vinylidene complex.

To conclude, one can anticipate continuing growth in the organometallic chemistry of propargylallenes, and look forward to reading the future chapters in its development.
